# Dendritic cells efficiently transmit HIV to T Cells in a tenofovir and raltegravir insensitive manner

**DOI:** 10.1371/journal.pone.0189945

**Published:** 2018-01-02

**Authors:** Jocelyn T. Kim, Emery Chang, Alex Sigal, David Baltimore

**Affiliations:** 1 Division of Biology and Biological Engineering, California Institute of Technology, Pasadena, California, United States of America; 2 Division of Infectious Diseases, Department of Medicine at University of California Los Angeles, Los Angeles, California, United States of America; 3 KwaZulu-Natal Research Institute for TB-HIV, Durban, South Africa; 4 University of KwaZulu-Natal, Durban, South Africa; 5 Max Planck Institute for Infection Biology, Berlin, Germany; University of Liverpool Institute of Infection and Global Health, UNITED KINGDOM

## Abstract

Dendritic cell (DC)-to-T cell transmission is an example of infection in *trans*, in which the cell transmitting the virus is itself uninfected. During this mode of DC-to-T cell transmission, uninfected DCs concentrate infectious virions, contact T cells and transmit these virions to target cells. Here, we investigated the efficiency of DC-to-T cell transmission on the number of cells infected and the sensitivity of this type of transmission to the antiretroviral drugs tenofovir (TFV) and raltegravir (RAL). We observed activated monocyte-derived and myeloid DCs amplified T cell infection, which resulted in drug insensitivity. This drug insensitivity was dependent on cell-to-cell contact and ratio of DCs to T cells in coculture. DC-mediated amplification of HIV-1 infection was efficient regardless of virus tropism or origin. The DC-to-T cell transmission of the T/F strain CH077.t/2627 was relatively insensitive to TFV compared to DC-free T cell infection. The input of virus modulated the drug sensitivity of DC-to-T cell infection, but not T cell infection by cell-free virus. At high viral inputs, DC-to-T cell transmission reduced the sensitivity of infection to TFV. Transmission of HIV by DCs in trans may have important implications for viral persistence *in vivo* in environments, where residual replication may persist in the face of antiretroviral therapy.

## Introduction

Cell-to-cell transmission of HIV describes a mechanism of viral transfer between a donor immune cell and target T cell [1–3] that involves a physical connection such as a virologic synapse [4, 5]. This efficiency is based upon the ability of the virus to exploit a physiologically occurring phenomenon, in which immune cells form synapses with one another. The infected donor cell uses the virologic synapse as a physical connection to deliver a high number of virions to an uninfected target cell[6–8]. Due to the high multiplicity of infection, HIV cell-to-cell spread has also been shown to be insensitive to certain antiretroviral drugs such as reverse transcriptase inhibitors [9–13]. A high multiplicity of infection can create an inflammatory environment through pyroptosis of bystander cells[14]. In addition, cell-to-cell transmission efficiently infects T cells in vivo[15, 16]. The high multiplicity of infection accelerates the onset of cellular infection[17] and increases the frequency of viral recombination, resulting in increased viral diversity[18, 19].

Cell-to-cell spread of HIV can occur between different cell types. Infected macrophages have been shown to efficiently transmit virions to T cells via cell-to-cell spread[7, 11]. In addition, cell-to-cell transmission also occurs between primary DCs and CD4^+^ T cells[1, 4]. Infection of mature DCs is rare due to host innate restriction factors such as SAMHD1[20]. However, infection of mature DCs is not required because DCs capture infectious virions and transmit them to uninfected target T cells via *trans*-infection[6, 21, 22]. Capture of the virus occurs through binding to various surface receptors such as DC-SIGN[23]. In addition, other surface receptors on DCs may play a role[24–27]. This transmission is inhibited by fusion inhibitors, but provides resistance to some HIV neutralizing antibodies[9, 28, 29]. One report demonstrates productively infected DCs can transmit HIV to T cells in the presence of protease inhibitors[28].

In this study we asked whether DC-to-T cell transmission in trans was efficient enough to reduce sensitivity to antiretroviral drugs in a similar way to cis infection. We observed that human monocyte-derived DCs (moDCs) amplified HIV infection of autologous peripheral blood mononuclear cells (PBMCs) and isolated primary CD4^+^ T cells. DC amplification of T cell infection resulted in relative insensitivity to the reverse transcriptase inhibitor TFV and integrase inhibitor RAL. Despite increasing the ratio of T cell targets to DCs, moDCs continued to efficiently transmit HIV in a drug-insensitive manner. Primary myeloid DCs also transmitted HIV to CD4^+^ T cells in a drug-insensitive manner, albeit with less efficiency compared to moDCs. We also found that DCs amplified infection of CXCR4 and CCR5-tropic isolates including transmitted founder (T/F) strains. DC-to-T cell transmission of the T/F strain CH077.t/2627 was insensitive to TFV. Lastly, we found drug insensitivity of DC-to-T cell infection was highly dependent on the virus input.

## Methods

### Culture of primary DCs, CD4^+^ T cells, and PBMCs

PBMCs were purified from whole blood (UCLA Center for AIDS Research Virology Core Lab) with a Ficoll-Paque Plus gradient. Following this purification, CD14^+^ peripheral blood monocytes were isolated using RosetteSep human monocyte enrichment cocktail (Stemcell Technologies). All primary cells were cultured in RPMI-1640 medium supplemented with 10% (vol/vol) fetal bovine serum (FBS) (Sigma), 1% (vol/vol) non-essential amino acids (HyClone), 1mM sodium pyruvate (Gibco), 10mM HEPES (Gibco), and 0.05mM 2-mercaptoethanol (Gibco). PBMCs at 1–2 × 10^6^ ml^-1^ were cultured in RPMI-based media with 20 μg ml^-1^ of human recombinant IL-2. Human CD14^+^ peripheral blood monocytes at 1 × 10^6^ cells ml^-1^ were cultured for 7 d in RPMI-based media containing 50ng ml^-1^ of human recombinant GM-CSF and 100ng ml^-1^ of human recombinant IL-4 (Peprotech) to generate human monocyte-derived DCs. DC media was replaced with new RPMI-based media containing fresh cytokines every 3 d. Primary CD4^+^ T cells were isolated from PBMCs using CD4 Microbeads (Miltenyi) and cultured in IL-2 containing media. Primary myeloid DCs were isolated from PBMCs using myeloid DC isolation kit (Miltenyi) and cultured in the GM-CSF and IL-4 containing media.

### Virus production

Molecular clones to generate infectious HIV-1 were obtained (AIDS Reagent Program, Division of AIDS, NIAID, NIH) (**S1 Table**). The NFN-SX isolate was a gift from the An Laboratory (UCLA). HEK293T/17 cells were plated in 10-cm tissue culture dishes and transfected with BioT (Bioland Scientific, Paramount CA) according to manufacturer's instructions using a total of 10 μg DNA. All viral supernatants were harvested at 48 h post-transfection and filtered through a 0.45-μm filter. The concentration of gag was measured by p24 capture ELISA Kit (ImmunoDiagnostics). Virus aliquots were stored at ^−^80°C.

### DC-T cell coculture

The moDCs at 1 × 10^6^ cells ml^-1^ at day 7 of culture and primary myeloid DCs at day 1 of culture were generated from the same donor and stimulated with 100ng ml^-1^ of Lipopolysaccharides LPS (Sigma-Aldrich) for 24 h. In parallel, autologous PBMCs or CD4^+^ T cells were activated with 5ng ml^-1^ of phytohemagglutinin (PHA) (Sigma-Aldrich) for 3 d. The DCs (moDCs or myeloid DCs) and T cell targets (PBMCs or CD4^+^ T cells) were washed with fresh media to remove LPS or PHA. DCs at 1 × 10^6^ cells ml^-1^ were cultured with or without PHA-activated PBMCs or CD4^+^ T cells at 1–2 × 10^6^ × 10^6^ cells ml^-1^ in varying donor-to-target ratios. In experiments using TFV or RAL (NIH AIDS Reagent Program), PBMCs or CD4^+^ T cells were first incubated with TFV or RAL for 6 h prior to coculture with DCs. Virus supernatant comprising 1/5 of the total volume was added to each well. In experiments involving transwell plates, PBMCs were seeded a 12-mm transwell plate (Corning). DCs and virus supernatant were added to the top of the 3.0-μm pore polycarbonate transwell membrane. Cells were cultures at for 2 d at 37°C.

### Flow cytometry analysis

Cells were stained for cell surface marker with the appropriate antibodies (**S2 Table**). For dead cell staining, 1ug ml^-1^ of propidium iodine was added. HIV infection was quantified based on HIV gag protein production measured by intracellular anti-p24 staining. Cells were permeabilized with PBS containing 1% bovine serum albumin BSA and 0.05% saponin and intracellular staining with anti-gag p24 monoclonal antibody (**S2 Table**). Flow cytometry data was acquired on a MACSQuant analyzer (Miltenyi). Data was analyzed with FlowJo software (TreeStar).

### Statistical analyses

GraphPad Prism 6.0 software was used for data analysis. Statistical significance was determined by unpaired, two-sided Student’s *t*-tests for two groups.

## Results

### moDCs amplify HIV infection of PBMCs

**Fig 1:**
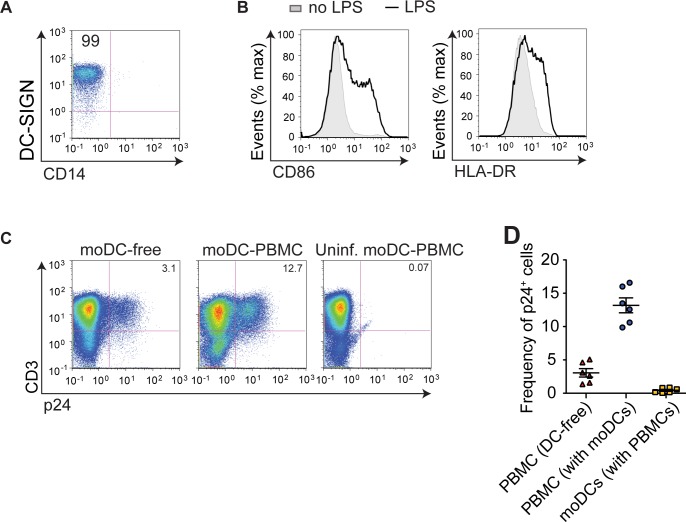
moDCs significantly amplify PBMC infection. (**A**, **B**) moDCs were treated with LPS and analyzed by flow cytometry. FACS histogram plots showing expression of DC-SIGN and CD14. Number in the top left gate indicates percentage of CD14^-^DC-SIGN^+^cells (**A**). FACS histogram plots showing surface expression of activation markers CD86 and HLA-DR (**B**). (**C**) Infection of PBMCs without (*left*) or with DCs (*center*) was analyzed by flow cytometry. Uninfected moDC-PBMC coculture (*right*) was included as a negative control. Number in the top right gate indicates percentage of infected T cells. (**D**) Frequency of infected p24^+^ T cells or moDCs was measured in DC-free culture or moDC-PBMC cell coculture. Each symbol represents one donor. Mean ± s.e.m (η = 6 donors). ***, p <0.0001 (student’s T-test). Data is representative of two independent (**A**, **B**), six independent (**C**) or pooled from six independent experiments (**D**).

To determine the effect of DCs on HIV infection of T cells, we infected PBMCs with the HIV-1 isolate NL4-3 in the presence or absence of autologous human moDCs at a DC-to-PBMC ratio of 1:1. The moDCs were matured with LPS and expressed the DC marker, DC-SIGN, as well as surface activation markers CD86 and HLA-DR (**Fig 1A and 1B**). To measure T cell infection, PBMCs were analyzed after a single round of infection (2 days of culture). We defined DC amplification of infection as the ratio of the frequency of infection in moDC-to-PMBC culture to frequency of infection in DC-free PBMC culture. During a single round of infection, we found moDCs amplified PBMC infection by two to five-fold depending on donor variability (**Fig 1C and 1D**). The frequency of infected moDCs in DC-PBMC cell coculture was low at approximately 0.14% (**Fig 1D**). This was consistent with previous reports that LPS-matured DCs capture significant numbers of HIV-1 particles and transmit them to T cells via *trans*-infection[22, 30].

### HIV transmission between moDCs and PBMCs is insensitive to TFV

**Fig 2:**
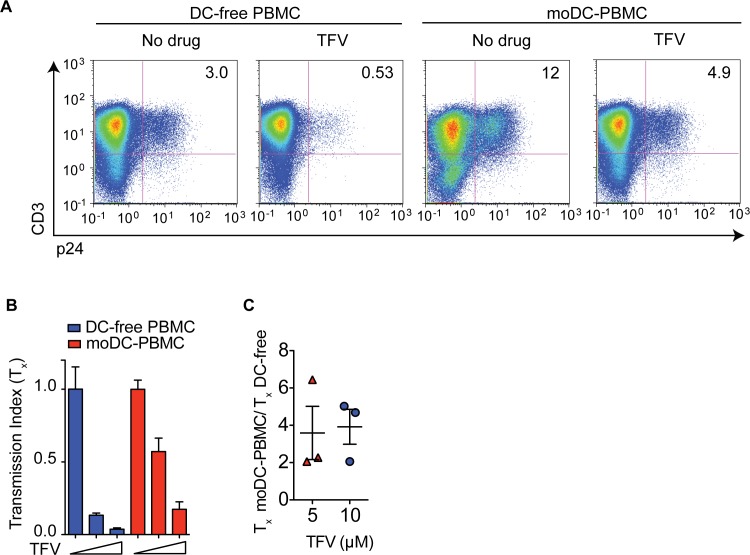
moDC-to-PBMC infection is insensitive to TFV. (**A**) FACS plots of DC-free T cell or moDC-to-PBMC infection in the absence of presence of 5 μM of TFV. (**B**) Drug insensitivity measured by transmission index (T_x_) of DC-free culture or moDC-T cell coculture. TFV at 0 **μ**M, 5 μM, 10 μM (wedges). (**C**) Fold difference between the T_x_ values of moDC-PBMC and DC-free PBMC infections. Each symbol represents one donor. Mean ± s.e.m (η = 3 donors). Data is representative of two independent experiments (**A**-**C**).

We next asked whether DC amplification of T cell infection results in antiretroviral drug insensitivity. We compared infection of DC-free PBMC culture and moDC-PBMC cell coculture in the presence or absence of the reverse transcriptase inhibitor TFV. From one donor, we found that T cell infection decreased 6-fold in the moDC-free culture compared to infection in moDC-PBMC coculture, which dropped approximately 2.5 fold (**Fig 2A**). We previously quantified sensitivity to drugs using the transmission index (T_x_), which is the fraction of T cells infected in the presence of drug divided by the fraction of T cells infected in the absence of drug[10]. A T_x_ value close to 1 indicates a high level of drug insensitivity and a T_x_ value <<1 indicates drug sensitivity. Comparison of the T_x_ values of DC-to-T cell infection and DC-free infection describes the relative drug insensitivity between the two modes of infection. T cell infection in moDC-PBMC coculture was 2 to 6-fold more insensitive to TFV compared to infection in DC-free PBMC culture (**Fig 2B and 2C**). This drug insensitivity of moDC-PBMC infection persisted despite increasing the dose of TFV (**Fig 2C**). These results are consistent with previous reports that reverse transcriptase inhibitors such as tenofovir do not effectively inhibit T cell-to-T cell infection[10, 13].

### moDC-to-PBMC transmission occurs via *trans* infection

**Fig 3:**
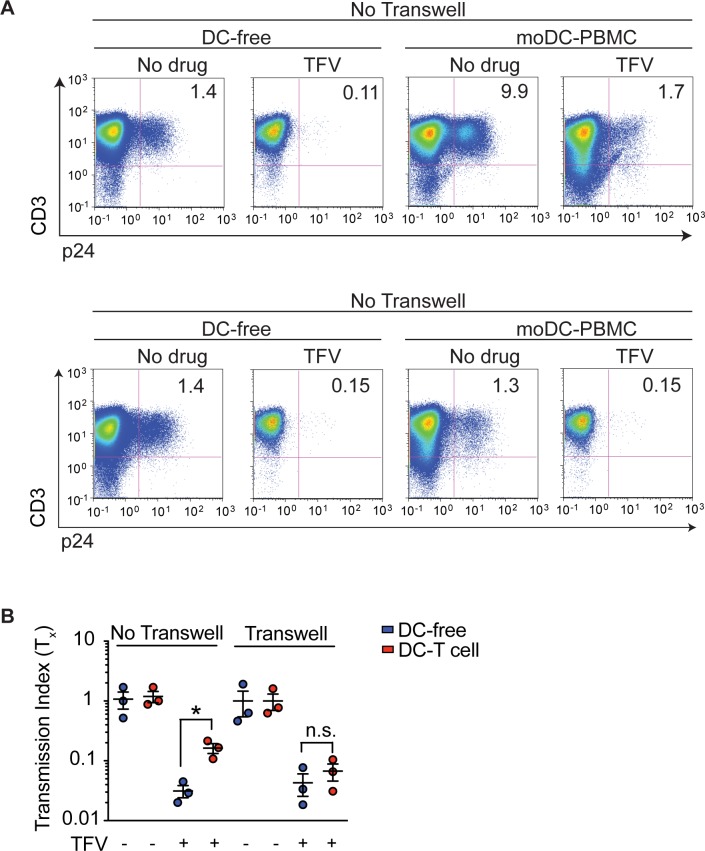
moDC-to-PBMC drug resistance depends on physical contact between cells. (**A**) FACS plots of DC-free PBMC and moDC-to-PBMC infection with (*bottom*) or without (*top*) a transwell system, in which DCs are physically separated from PBMCs by a transwell membrane. Infection occurs in the absence of presence of 10 μM of TFV. (**B**) Drug insensitivity (T_x_) of DC-free or moDC-to-PBMC infection with or without 10 μM of TFV and in the absence of presence of a transwell system. Mean ± s.e.m (η = 3 donors). *, p <0.05 (student’s T-test). n.s., p >0.05. Each symbol represents a donor. Data is representative of two (**A**, **B**) independent experiments.

Since HIV cell-to-cell spread occurs via close proximity such as a virologic synapse, we next sought to determine whether the efficiency of moDC-to-PBMC transmission was completely dependent on physical contact or whether secreted factors from DCs modulate infection. DC amplification of T cell infection was abolished if moDCs and PBMCs were physically separated by a transwell membrane (**Fig 3A**). In addition, we found moDC-to-PBMC cell drug insensitivity was dependent on physical contact between the DCs and T cell targets (**Fig 3A and 3B**). These data suggest drug-insensitivity of DC-to-T cell infection was dependent on the ability of DCs to concentrate and transmit virions to T cell through physical interaction and not because of factors secreted by DCs. These results are also consistent with previous results that DCs amplify infection in a contact-dependent manner[5].

### HIV transmission between moDCs and T cells is efficient and insensitive to RAL

**Fig 4:**
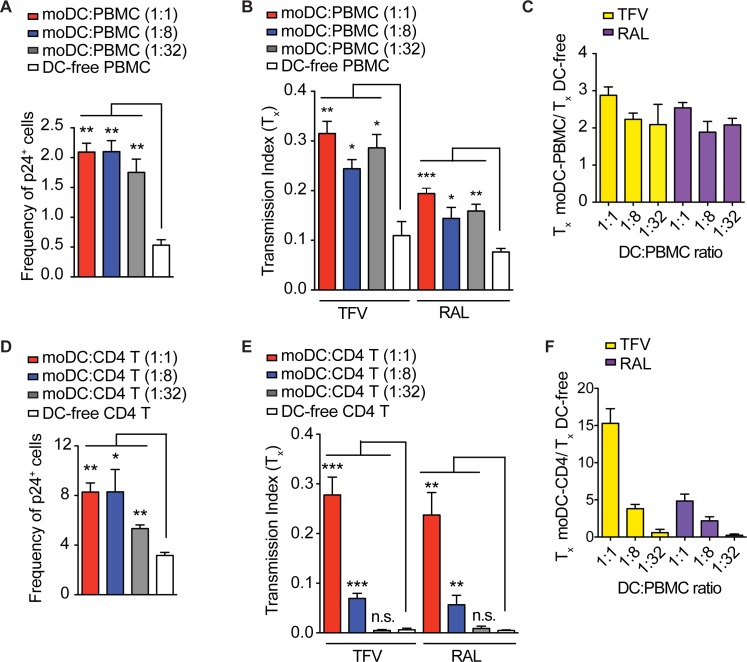
HIV transmission between moDCs and PBMCs or isolated CD4^+^ T cells is efficient and insensitive to RAL. (**A, D**) The moDCs were cocultured with autologous PBMCs (A) or CD4^+^ T cells (D) at ratios of 1:1, 1:8, and 1:32. The cultures were infected with NL4-3. The frequency of infected p24^+^ T cells was measured using flow cytometry. (**B, E**) The moDC-PBMC coculture (B) or moDC-CD4^+^ T cell coculture (E) or DC-free cultures were infected with NL4-3 in the presence or absence of 10 μM of TFV or 10 μM of RAL and drug insensitivity (T_x_) was measured. (**C, F**) Fold differences between the T_x_ values of moDC-PBMC coculture (C) or moDC-CD4^+^ T cell coculture (F) and DC-free culture were measured. Mean ± s.e.m (η = 3 technical replicates). *, p <0.05; **, p <0.01, ***, p<0.005 (student’s T-test). n.s., p >0.05. Data is representative of one donor from two independent experiments (**A**-**F**).

To evaluate the efficiency of transmission between moDCs and PBMCs, we infected cocultures of moDCs and PBMCs and found that moDCs continued to amplify T cell infection even at the DC-to-PBMC ratio of 1:32 (**Fig 4A**). At moDC-to-PBMC ratios ranging between 1:1 and 1:32, we found moDC-to-PBMC infection continued to be at least 2-fold more insensitive to TFV compared to DC-free PBMC infection (**Fig 4B and 4C**).

The reverse transcriptase inhibitors, NRTIs and NNRTIs, have been less effective against cell-to-cell transmission compared to entry and protease inhibitors[5, 7, 9–13]. We next questioned whether integrase inhibitors could effectively inhibit cell-to-cell transmission given that they have been shown to prevent pyroptosis mediated by cell-to-cell infection[14]. Similar to its TFV insensitivity, we found that moDC-to-PBMC infection was 2 to 2.5-fold more insensitive to RAL compared to DC-free PBMC infection (**Fig 4B and 4C**). The RAL insensitivity of moDC-to-PBMC infection was observed even at the moDC-to-PBMC ratio of 1:32. These results suggest that moDCs efficiently transmit HIV to T cell targets, which allows for a TFV and RAL-insensitive mode of infection.

We next assessed whether HIV transmission between moDCs and enriched primary CD4^+^ T cells was efficient. We found the frequency of T cell infection was higher in moDC-CD4^+^ T cell cocultures compared to moDC-PBMC cocultures (**Fig 4A and 4D**), which is likely due to the inhibitory effect of CD8^+^ T cells on CD4^+^ T cell infection [31, 32]. The moDCs cocultured with CD4^+^ T cells at ratios of 1:1 and 1:8 efficiently amplifed infection by 2.6-fold (**Fig 4D**), which resulted in TFV and RAL insensitivity to moDC-to-CD4^+^ T cell transmission (**Fig 4E**). We found moDC-to-CD4^+^ T cell infection was at least 2-fold more insensitive to TFV or RAL at moDC-to-CD4^+^ T cell ratios of 1:1 and 1:8, but not 1:32 (**Fig 4F**). These data demonstrate moDCs transmit HIV to CD4^+^ T cells in a relatively TFV and RAL insensitive manner.

### HIV transmission between primary myeloid DCs and CD4^+^ T cells is insensitive to TFV

**Fig 5:**
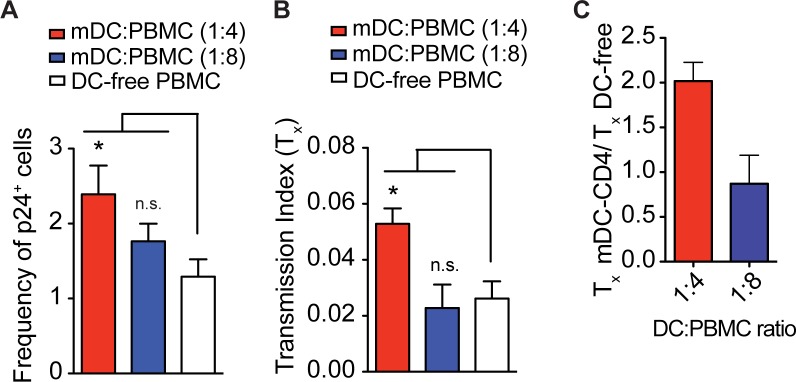
HIV transmission between primary myeloid DCs and CD4^+^ T cells is capable of drug-insensitivity. Primary myeloid DCs (mDCs) were cocultured with autologous CD4^+^ T cells at ratios of 1:4 and 1:8. (**A**) The mDC-CD4^+^ T cell cocultures and DC-free CD4^+^ T cell cultures were infected with NL4-3 and the frequency of infected p24^+^ T cells was measured using flow cytometry. (**B**) The mDC-CD4^+^ T cell cocultures and DC-free CD4^+^ T cell cultures were infected with NL4-3 in the presence or absence of 10 μM of TFV and drug insensitivity (T_x_) was measured. (**C**) Fold difference between the T_x_ values of mDC-CD4^+^ T cell coculture and DC-free culture were measured. Mean ± s.e.m (η = 3 technical replicates). *, p <0.05 (student’s T-test); n.s., p >0.05. Data is representative of one donor from two independent experiments (**A**-**C**).

We then assessed the efficiency of primary myeloid DCs transmitted HIV to CD4^+^ T cells. Prior work has found that immature myeloid DCs are capable of infecting T cells via cis and trans-infection[33]. Indeed, mature myeloid DCs are more efficient at capturing and transmitting HIV to T cell lines[34]. To assess the efficiency of DC-to-T cell transmission, we isolated primary myeloid DCs from the peripheral blood, stimulated them with LPS, and cocultured them with autologous CD4^+^ T cells at DC-to-T cell ratios of 1:4 and 1:8. The myeloid DCs amplified T cell infection by approximately 2-fold in cocultures with myeloid DC-to-CD4^+^ T cell ratio of 1:4, but not 1:8 **(Fig 5A).** We also observed that myeloid DCs and CD4^+^ T cells cocultured at a ratio of 1:4 demonstrated approximately 2-fold insensitivity to TFV compared to DC-free CD4^+^ T cell culture (**Fig 5B and 5C**). In addition, we compared HIV infection of CD4^+^ T cocultured with autologous moDCs and myeloid DCs. The myeloid DCs were less efficient than moDCs in transmitting HIV to CD4^+^ T cells (**Figs 4D and 5A**). The myeloid DCs may be less efficient at trans-infection due to decreased expression of C-type lectins such as DC-SIGN that are important to viral capture (S1 Fig) [34, 35]. However, there are likely other surface molecules expressed on activated myeloid DCs such as Siglec-1, which allow for virus capture[36].

### DCs efficiently amplify infection with transmitted/founder strains

**Fig 6:**
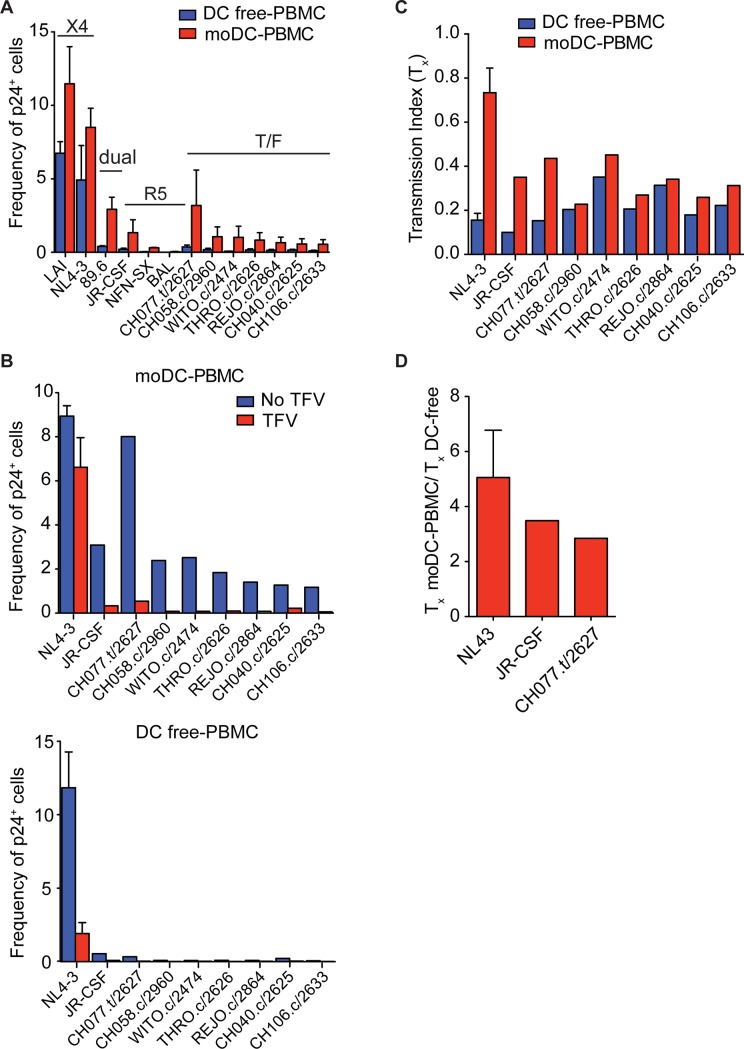
DC amplification of T cell infection with different HIV-1 isolates. (**A**) Infection of PBMCs with or without moDCs by CXCR4-tropic, dual-tropic, CCR5-tropic and T/F isolates, which were normalized to similar amounts of p24, were analyzed by flow cytometry. Frequency of p24^+^ T cells was measured by flow cytometry. (**B, C**) PBMCs with or without moDCs were infected with or without 10 μM of TFV and drug insensitivity (T_x_) (B) and fold difference between the T_x_ values were measured (C). Mean ± s.e.m (η = 2 donors). Data is representative of three independent experiments (**A-C**).

Since DCs could be particularly important in the initial amplification of T cell infection when a patient is first infected, we next sought to investigate whether CCR5-tropic transmitted/founder (T/F) strains are also effectively transmitted by DCs to T cells. Recent work suggested that CCR5-tropic T/F strains are more infectious due to increased Env expression on the virions and efficient binding to DCs[37]. Despite the low frequency of infection in DC-free cultures, moDCs efficiently amplified infection of the T/F strains (**Fig 6A**). Thus, the effect of TFV on moDC-to-PBMC infection resulted in a fraction of T cells insensitive to TFV (T_x_) ranging between 0.2 to 0.4 (**Fig 6B**, *top*, **and 6C**). The overall frequency of DC-free PBMC infection by T/F strains was less than 0.1%, which approached the level of background staining at 0.07% (**Figs 6A and 1C**). Thus, the addition of TFV to DC-free cultures did not have a large inhibitory effect on transmission of T/F strains (**Fig 6B,** *bottom***, and 6C**). However, the T/F strain CH077.t/2627 infected PBMCs at a similar frequency to the laboratory-adapted strain JR-CSF (**Fig 6A**). Thus, we found DC-to-PBMC transmission with the T/F strain CHO77.t/2627 was approximately 3-fold more insensitive to TFV compared to DC-free infection (**Fig 6D**). Altogether, we found DCs were able to amplify T cell infection efficiently with T/F strains. In particular, DC-to-PBMC transmission with the more infectious T/F strain CHO77.t/2627 was insensitive to TFV.

### Virus input determines the sensitivity of DC-to-T cell transmission to TFV

**Fig 7:**
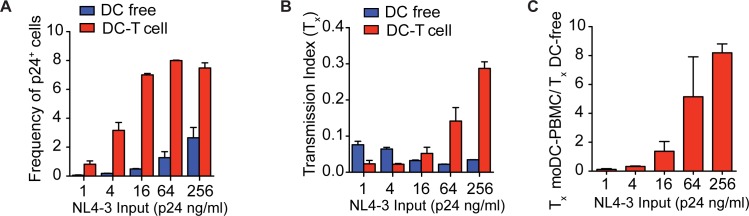
TFV insensitivity of moDC-to-PBMC transmission is dependent on infectious dose of NL4-3. (**A**) PBMCs with or without moDCs were infected with varying doses of NL4-3. Frequency of p24^+^ T cells was measured by flow cytometry. (**B, C**) PBMCs with or without moDCs were infected with varying doses of NL4-3 in the presence or absence of 10 μM of TFV and drug insensitivity (T_x_) (B) and fold difference between the T_x_ values were measured (C). Mean ± s.e.m (η = 2 donors). Data is representative of two independent experiments (**A-C**).

We suspected that drug insensitivity was dependent on varying concentrations of infectious particles per ng of p24. To test this hypothesis, we decreased the NL4-3 input such that the frequency of DC-free infection was similar to that of the CCR5-tropic isolates. At higher levels of NL4-3 input, DC-to-T cell transmission plateaued at approximately 8% suggesting that this mode of transmission had saturated the infectable T cell population (**Fig 7A**), which was consistent with a high multiplicity of infection. Therefore, at the higher levels of NL4-3 input, drug insensitivity (T_x_) was higher in DC-to-PBMC transmission compared to DC-free infection because DCs transmitted a high MOI (**Fig 7B and 7C**). With more virus input, DC-to-T cell transmission allows multiple infectious virions to infect one T cell, producing the relative drug insensitivity. As input levels of NL4-3 decreases, DC-to-T infection became more sensitive to TFV. At the lower levels of NL4-3 input, drug insensitivity (T_x_) was higher with DC-free infection compared to moDC-PBMC infection. This result is due to the fact that the frequency of DC-free infection was less than 0.2%, which was also observed with most of the T/F strains. The addition of TFV did not have a large inhibitory effect because the initial number of infected T cells was already low.

## Discussion

Cell-to-cell transmission between an infected donor cell and an uninfected T cell target yields a high multiplicity of infection per target cell, which results in insensitivity to certain antiretroviral drugs [10, 11, 28, 33]. In this report, we examined cell-to-cell transmission of HIV between primary DCs and T cells and found such transmissions amplified the multiplicity of infection, resulting in insensitivity to TFV and RAL. We found DC amplification of T cell infection was entirely dependent on cell-to-cell contact, which was consistent with trans infection[5, 38]. These results are explained by the ability of DCs to efficiently capture virions and direct them to T cell targets, which leads to amplification of T cell infection, locally high multiplicity of infection, and subsequent antiretroviral drug insensitivity. Also, we found that activated myeloid DCs transmitted virus in a drug-insensitive manner, which is consistent with prior work demonstrating that myeloid DCs can capture virus[36].

We also compared DC-to-T cell transmission by T/F strains and laboratory-adapted CXCR4 and CCR5-tropic strains and found this mode of infection was efficient in amplifying infection by all the strains. Others have compared T/F strains to CCR5-tropic control strains isolated from patients with chronic infection and found that these T/F strains have increased infectivity in single round of infection assays, higher concentrations of Env per particle and increased binding capacity to DCs[37]. This could explain why the T/F strains were as efficient as the laboratory-adapted strains at DC-to-T cell transmission. Because the frequency of T cells infected by T/F strains in DC-free cultures was close to background staining levels, the full effect of TFV in these cultures was difficult to determine. Attempts to increase the infectivity of the T/F strains by ultracentifugation were not successful. However, we found one of the T/F strains CH077.t/2627 was more infectious and resulted in TFV insensitivity of DC-to-PBMC transmission relative to DC-free infection.

Our results with the T/F strains suggested that the infectivity of the virus was important to DC-to-T cell transmission. Indeed, we found drug insensitivity of DC-to-T cell transmission increased as the amount of virus added to coculture was increased. These results indicate sufficient virions must be available for DCs to deliver enough copies per cell to obtain some degree of insensitivity to antiretroviral drugs. In addition, there must be sufficient virions capable of infecting T cells to assess the full effect of drugs in DC-free cultures. These results suggest DC-to-T cell transmission may result in reduced sensitivity to drugs under conditions of high virus concentration. This highlights an important difference relative to infection in cis. For example, in T cell-T cell transmission the infected donor T cell is a local source of the high number of virions transmitted to the target T cell producing drug insensitivity compared to cell-free infection[10, 39]. In comparison, mature DCs are rarely infected with HIV[22], and thus transmission depends on the number of viral particles nearby available for capture.

Our findings have implications for the importance of DCs in HIV infection. During initial infection, DCs are among the first immune cells to encounter HIV at mucosal surfaces[21]. Upon maturation, DCs carrying virions migrate to lymphoid organs[40, 41] where DC-to-T cell transmission may play an important role in spreading the virus[21]. Given that the probability of successful infection per HIV exposure is low, DCs may increase the probability of establishing infection not only by transporting the virus, but also by amplifying the number of cells the viral input can infect. Our results indicate that this amplification may be as high as 5-fold with one cycle of transmission, which should strongly influence the chance of early infection to persist.

Importantly, this study shows that *trans*-infection from DCs to T cells can result in reduced sensitivity to certain antiretroviral drugs such as TFV and RAL. Such transmission also occurs between follicular dendritic cells and follicular T helper cells in the germinal center[42, 43]. Recent studies observed extensive infection of the PD-1^+^, CXCR5^+^ follicular T helper cell subset[44–46]. Whether such transmission can take place in the lymph node environment in the face of antiretroviral therapy is controversial[7, 9, 11–13, 47–49]. However, given the possibility of lower drug penetrance in lymph nodes[50, 51] and ability of follicular DCs to retain intact virus despite ART[43] and preferentially transmit to antigen-specific T cells[33], DC *trans*-infection has the potential to play a part in persistence, and further studies are needed to determine its role *in vivo*.

## Supporting information

S1 Fig DC-SIGN and HLA-DR expression of LPS-activated myeloid mDCs. (PDF)

S1 Table HIV-1 molecular clones. (PDF)

S2 Table Antibodies used in this study. (PDF)

## References

[1] McDonald D, Wu L, Bohks SM, KewalRamani VN, Unutmaz D, Hope TJ. Recruitment of HIV and its receptors to dendritic cell-T cell junctions. Science. 2003;300(5623):1295–7. doi: 10.1126/science.1084238 . 12730499 10.1126/science.1084238

[2] Jolly C, Kashefi K, Hollinshead M, Sattentau QJ. HIV-1 cell to cell transfer across an Env-induced, actin-dependent synapse. J Exp Med. 2004;199(2):283–93. doi: 10.1084/jem.20030648 ; PubMed Central PMCID: PMCPMC2211771. 14734528 10.1084/jem.20030648 PMC2211771

[3] Groot F, Welsch S, Sattentau QJ. Efficient HIV-1 transmission from macrophages to T cells across transient virological synapses. Blood. 2008;111(9):4660–3. doi: 10.1182/blood-2007-12-130070 . 18296630 10.1182/blood-2007-12-130070

[4] Felts RL, Narayan K, Estes JD, Shi D, Trubey CM, Fu J, et al 3D visualization of HIV transfer at the virological synapse between dendritic cells and T cells. Proc Natl Acad Sci U S A. 2010;107(30):13336–41. doi: 10.1073/pnas.1003040107 ; PubMed Central PMCID: PMCPMC2922156. 20624966 10.1073/pnas.1003040107 PMC2922156

[5] Martin N, Welsch S, Jolly C, Briggs JAG, Vaux D, Sattentau QJ. Virological Synapse-Mediated Spread of Human Immunodeficiency Virus Type 1 between T Cells Is Sensitive to Entry Inhibition. Journal of virology. 2010;84(7):3516–27. doi: 10.1128/JVI.02651-09. WOS:000275307400036. 20089656 10.1128/JVI.02651-09 PMC2838118

[6] Cameron PU, Freudenthal PS, Barker JM, Gezelter S, Inaba K, Steinman RM. Dendritic cells exposed to human immunodeficiency virus type-1 transmit a vigorous cytopathic infection to CD4+ T cells. Science. 1992;257(5068):383–7. . 1352913 10.1126/science.1352913

[7] Carr JM, Hocking H, Li P, Burrell CJ. Rapid and efficient cell-to-cell transmission of human immunodeficiency virus infection from monocyte-derived macrophages to peripheral blood lymphocytes. Virology. 1999;265(2):319–29. doi: 10.1006/viro.1999.0047 . 10600603 10.1006/viro.1999.0047

[8] Zhong P, Agosto LM, Ilinskaya A, Dorjbal B, Truong R, Derse D, et al Cell-to-cell transmission can overcome multiple donor and target cell barriers imposed on cell-free HIV. PLoS One. 2013;8(1):e53138 doi: 10.1371/journal.pone.0053138 ; PubMed Central PMCID: PMCPMC3538641. 23308151 10.1371/journal.pone.0053138 PMC3538641

[9] Gupta P, Balachandran R, Ho M, Enrico A, Rinaldo C. Cell-to-cell transmission of human immunodeficiency virus type 1 in the presence of azidothymidine and neutralizing antibody. Journal of virology. 1989;63(5):2361–5. ; PubMed Central PMCID: PMCPMC250658. 2704079 10.1128/jvi.63.5.2361-2365.1989 PMC250658

[10] Sigal A, Kim JT, Balazs AB, Dekel E, Mayo A, Milo R, et al Cell-to-cell spread of HIV permits ongoing replication despite antiretroviral therapy. Nature. 2011;477(7362):95–8. doi: 10.1038/nature10347 . 21849975 10.1038/nature10347

[11] Duncan CJ, Russell RA, Sattentau QJ. High multiplicity HIV-1 cell-to-cell transmission from macrophages to CD4+ T cells limits antiretroviral efficacy. Aids. 2013;27(14):2201–6. doi: 10.1097/QAD.0b013e3283632ec4 ; PubMed Central PMCID: PMCPMC4714465. 24005480 10.1097/QAD.0b013e3283632ec4 PMC4714465

[12] Agosto LM, Zhong P, Munro J, Mothes W. Highly Active Antiretroviral Therapies Are Effective against HIV-1 Cell-to-Cell Transmission. PLoS pathogens. 2014;10(2). ARTN e1003982 doi: 10.1371/journal.ppat.1003982. WOS:000332085900053. 24586176 10.1371/journal.ppat.1003982 PMC3937346

[13] Titanji BK, Aasa-Chapman M, Pillay D, Jolly C. Protease inhibitors effectively block cell-to-cell spread of HIV-1 between T cells. Retrovirology. 2013;10 Artn 161 doi: 10.1186/1742-4690-10-161. WOS:000329050400001. 24364896 10.1186/1742-4690-10-161 PMC3877983

[14] Galloway NLK, Doitsh G, Monroe KM, Yang ZY, Munoz-Arias I, Levy DN, et al Cell-to-Cell Transmission of HIV-1 Is Required to Trigger Pyroptotic Death of Lymphoid-Tissue-Derived CD4 T Cells. Cell Rep. 2015;12(10):1555–63. doi: 10.1016/j.celrep.2015.08.011. WOS:000360965500005. 26321639 10.1016/j.celrep.2015.08.011 PMC4565731

[15] Kolodkin-Gal D, Hulot SL, Korioth-Schmitz B, Gombos RB, Zheng Y, Owuor J, et al Efficiency of cell-free and cell-associated virus in mucosal transmission of human immunodeficiency virus type 1 and simian immunodeficiency virus. Journal of virology. 2013;87(24):13589–97. doi: 10.1128/JVI.03108-12 ; PubMed Central PMCID: PMCPMC3838232. 24109227 10.1128/JVI.03108-12 PMC3838232

[16] Murooka TT, Deruaz M, Marangoni F, Vrbanac VD, Seung E, von Andrian UH, et al HIV-infected T cells are migratory vehicles for viral dissemination. Nature. 2012;490(7419):283–7. Epub 2012/08/03. doi: 10.1038/nature11398 [pii]. ; PubMed Central PMCID: PMC3470742. 22854780 10.1038/nature11398 PMC3470742

[17] Mikael Boulle TGM, Sabrina Dahling, Yashica Ganga, Laurelle Jackson DM, Lance Oom, Gila Lustig, Richard A. Neher, Sigal A. HIV Cell-to-Cell Spread Results in Earlier Onset of Viral Gene Expression by Multiple Infections per Cell. PLoS pathogens. 2016 doi: 10.1371/journal.ppat.1005964 27812216 10.1371/journal.ppat.1005964 PMC5094736

[18] Law KM, Komarova NL, Yewdall AW, Lee RK, Herrera OL, Wodarz D, et al In Vivo HIV-1 Cell-to-Cell Transmission Promotes Multicopy Micro-compartmentalized Infection. Cell Rep. 2016;15(12):2771–83. doi: 10.1016/j.celrep.2016.05.059 . 27292632 10.1016/j.celrep.2016.05.059

[19] Del Portillo A, Tripodi J, Najfeld V, Wodarz D, Levy DN, Chen BK. Multiploid inheritance of HIV-1 during cell-to-cell infection. Journal of virology. 2011;85(14):7169–76. doi: 10.1128/JVI.00231-11 ; PubMed Central PMCID: PMCPMC3126592. 21543479 10.1128/JVI.00231-11 PMC3126592

[20] Laguette N, Sobhian B, Casartelli N, Ringeard M, Chable-Bessia C, Segeral E, et al SAMHD1 is the dendritic- and myeloid-cell-specific HIV-1 restriction factor counteracted by Vpx. Nature. 2011;474(7353):654–U132. doi: 10.1038/nature10117. WOS:000292204300042. 21613998 10.1038/nature10117 PMC3595993

[21] Wu L, KewalRamani VN. Dendritic-cell interactions with HIV: infection and viral dissemination. Nature reviews Immunology. 2006;6(11):859–68. doi: 10.1038/nri1960 ; PubMed Central PMCID: PMCPMC1796806. 17063186 10.1038/nri1960 PMC1796806

[22] Dong C, Janas AM, Wang JH, Olson WJ, Wu L. Characterization of human immunodeficiency virus type 1 replication in immature and mature dendritic cells reveals dissociable cis- and trans-infection. Journal of virology. 2007;81(20):11352–62. doi: 10.1128/JVI.01081-07 ; PubMed Central PMCID: PMCPMC2045571. 17686876 10.1128/JVI.01081-07 PMC2045571

[23] Geijtenbeek TB, Kwon DS, Torensma R, van Vliet SJ, van Duijnhoven GC, Middel J, et al DC-SIGN, a dendritic cell-specific HIV-1-binding protein that enhances trans-infection of T cells. Cell. 2000;100(5):587–97. . 10721995 10.1016/s0092-8674(00)80694-7

[24] Gummuluru S, Rogel M, Stamatatos L, Emerman M. Binding of human immunodeficiency virus type 1 to immature dendritic cells can occur independently of DC-SIGN and mannose binding C-type lectin receptors via a cholesterol-dependent pathway. Journal of virology. 2003;77(23):12865–74. doi: 10.1128/JVI.77.23.12865-12874.2003. WOS:000186612700046. 14610207 10.1128/JVI.77.23.12865-12874.2003 PMC262553

[25] Lambert AA, Gilbert C, Richard M, Beaulieu AD, Tremblay MJ. The C-type lectin surface receptor DCIR acts as a new attachment factor for HIV-1 in dendritic cells and contributes to trans- and cis-infection pathways. Blood. 2008;112(4):1299–307. doi: 10.1182/blood-2008-01-136473 ; PubMed Central PMCID: PMCPMC2515113. 18541725 10.1182/blood-2008-01-136473 PMC2515113

[26] Magerus-Chatinet A, Yu HF, Garcia S, Ducloux E, Terris B, Bomsel M. Galactosyl ceramide expressed on dendritic cells can mediate HIV-1 transfer from monocyte derived dendritic cells to autologous T cells. Virology. 2007;362(1):67–74. doi: 10.1016/j.virol.2006.11.035. WOS:000246563300009. 17234232 10.1016/j.virol.2006.11.035

[27] Izquierdo-Useros N, Lorizate M, Puertas MC, Rodriguez-Plata MT, Zangger N, Erikson E, et al Siglec-1 Is a Novel Dendritic Cell Receptor That Mediates HIV-1 Trans-Infection Through Recognition of Viral Membrane Gangliosides. Plos Biol. 2012;10(12). ARTN e1001448 doi: 10.1371/journal.pbio.1001448. WOS:000312905300010. 23271952 10.1371/journal.pbio.1001448 PMC3525531

[28] Ganesh L, Leung K, Lore K, Levin R, Panet A, Schwartz O, et al Infection of specific dendritic cells by CCR5-tropic human immunodeficiency virus type 1 promotes cell-mediated transmission of virus resistant to broadly neutralizing antibodies. Journal of virology. 2004;78(21):11980–7. doi: 10.1128/JVI.78.21.11980-11987.2004. WOS:000224540900051. 15479838 10.1128/JVI.78.21.11980-11987.2004 PMC523246

[29] Ketas TJ, Frank I, Klasse PJ, Sullivan BM, Gardner JP, Spenlehauer C, et al Human immunodeficiency virus type 1 attachment, coreceptor, and fusion inhibitors are active against both direct and trans infection of primary cells. Journal of virology. 2003;77(4):2762–7. doi: 10.1128/JVI.77.4.2762-2767.2003 ; PubMed Central PMCID: PMCPMC141110. 12552019 10.1128/JVI.77.4.2762-2767.2003 PMC141110

[30] Izquierdo-Useros N, Lorizate M, McLaren PJ, Telenti A, Krausslich HG, Martinez-Picado J. HIV-1 capture and transmission by dendritic cells: the role of viral glycolipids and the cellular receptor Siglec-1. PLoS pathogens. 2014;10(7):e1004146 doi: 10.1371/journal.ppat.1004146 ; PubMed Central PMCID: PMC4102576. 25033082 10.1371/journal.ppat.1004146 PMC4102576

[31] Cocchi F, DeVico AL, Garzino-Demo A, Arya SK, Gallo RC, Lusso P. Identification of RANTES, MIP-1 alpha, and MIP-1 beta as the major HIV-suppressive factors produced by CD8+ T cells. Science. 1995;270(5243):1811–5. . 8525373 10.1126/science.270.5243.1811

[32] Tomaras GD, Lacey SF, McDanal CB, Ferrari G, Weinhold KJ, Greenberg ML. CD8+ T cell-mediated suppressive activity inhibits HIV-1 after virus entry with kinetics indicating effects on virus gene expression. Proc Natl Acad Sci U S A. 2000;97(7):3503–8. doi: 10.1073/pnas.070521097 ; PubMed Central PMCID: PMCPMC16269. 10725407 10.1073/pnas.070521097 PMC16269

[33] Lore K, Smed-Sorensen A, Vasudevan J, Mascola JR, Koup RA. Myeloid and plasmacytoid dendritic cells transfer HIV-1 preferentially to antigen-specific CD4+ T cells. J Exp Med. 2005;201(12):2023–33. doi: 10.1084/jem.20042413 ; PubMed Central PMCID: PMCPMC2212038. 15967828 10.1084/jem.20042413 PMC2212038

[34] Izquierdo-Useros N, Blanco J, Erkizia I, Fernandez-Figueras MT, Borras FE, Naranjo-Gomez M, et al Maturation of blood-derived dendritic cells enhances human immunodeficiency virus type 1 capture and transmission. Journal of virology. 2007;81(14):7559–70. doi: 10.1128/JVI.02572-06 ; PubMed Central PMCID: PMCPMC1933337. 17475656 10.1128/JVI.02572-06 PMC1933337

[35] Turville SG, Arthos J, Donald KM, Lynch G, Naif H, Clark G, et al HIV gp120 receptors on human dendritic cells. Blood. 2001;98(8):2482–8. . 11588046 10.1182/blood.v98.8.2482

[36] Puryear WB, Akiyama H, Geer SD, Ramirez NP, Yu X, Reinhard BM, et al Interferon-inducible mechanism of dendritic cell-mediated HIV-1 dissemination is dependent on Siglec-1/CD169. PLoS pathogens. 2013;9(4):e1003291 doi: 10.1371/journal.ppat.1003291 ; PubMed Central PMCID: PMCPMC3623718. 23593001 10.1371/journal.ppat.1003291 PMC3623718

[37] Parrish NF, Gao F, Li H, Giorgi EE, Barbian HJ, Parrish EH, et al Phenotypic properties of transmitted founder HIV-1. P Natl Acad Sci USA. 2013;110(17):6626–33. doi: 10.1073/pnas.1304288110. WOS:000318677300020. 23542380 10.1073/pnas.1304288110 PMC3637789

[38] Granelli-Piperno A, Finkel V, Delgado E, Steinman RM. Virus replication begins in dendritic cells during the transmission of HIV-1 from mature dendritic cells to T cells. Current biology: CB. 1999;9(1):21–9. . 9889121 10.1016/s0960-9822(99)80043-8

[39] Monel B, Beaumont E, Vendrame D, Schwartz O, Brand D, Mammano F. HIV cell-to-cell transmission requires the production of infectious virus particles and does not proceed through env-mediated fusion pores. Journal of virology. 2012;86(7):3924–33. doi: 10.1128/JVI.06478-11 ; PubMed Central PMCID: PMCPMC3302491. 22258237 10.1128/JVI.06478-11 PMC3302491

[40] Banchereau J, Steinman RM. Dendritic cells and the control of immunity. Nature. 1998;392(6673):245–52. doi: 10.1038/32588 . 9521319 10.1038/32588

[41] Mellman I, Turley SJ, Steinman RM. Antigen processing for amateurs and professionals. Trends Cell Biol. 1998;8(6):231–7. . 9695847 10.1016/s0962-8924(98)01276-8

[42] Spiegel H, Herbst H, Niedobitek G, Foss HD, Stein H. Follicular Dendritic Cells Are a Major Reservoir for Human-Immunodeficiency-Virus Type-1 in Lymphoid-Tissues Facilitating Infection of Cd4+ T-Helper Cells. Am J Pathol. 1992;140(1):15–22. WOS:A1992GZ63600004. 1530997 PMC1886262

[43] Heesters BA, Lindqvist M, Vagefi PA, Scully EP, Schildberg FA, Altfeld M, et al Follicular Dendritic Cells Retain Infectious HIV in Cycling Endosomes. PLoS pathogens. 2015;11(12):e1005285 doi: 10.1371/journal.ppat.1005285 ; PubMed Central PMCID: PMCPMC4666623. 26623655 10.1371/journal.ppat.1005285 PMC4666623

[44] Banga R, Procopio FA, Noto A, Pollakis G, Cavassini M, Ohmiti K, et al PD-1(+) and follicular helper T cells are responsible for persistent HIV-1 transcription in treated aviremic individuals. Nat Med. 2016;22(7):754–61. Epub 2016/05/31. doi: 10.1038/nm.4113 [pii]. . 27239760 10.1038/nm.4113

[45] Fukazawa Y, Lum R, Okoye AA, Park H, Matsuda K, Bae JY, et al B cell follicle sanctuary permits persistent productive simian immunodeficiency virus infection in elite controllers. Nat Med. 2015;21(2):132–9. Epub 2015/01/20. doi: 10.1038/nm.3781 [pii]. ; PubMed Central PMCID: PMC4320022. 25599132 10.1038/nm.3781 PMC4320022

[46] Boritz EA, Darko S, Swaszek L, Wolf G, Wells D, Wu X, et al Multiple Origins of Virus Persistence during Natural Control of HIV Infection. Cell. 2016;166(4):1004–15. doi: 10.1016/j.cell.2016.06.039 ; PubMed Central PMCID: PMCPMC4983216. 27453467 10.1016/j.cell.2016.06.039 PMC4983216

[47] Sigal A, Baltimore D. As good as it gets? The problem of HIV persistence despite antiretroviral drugs. Cell host & microbe. 2012;12(2):132–8. doi: 10.1016/j.chom.2012.07.005 . 22901535 10.1016/j.chom.2012.07.005

[48] Mathez D, Schinazi RF, Liotta DC, Leibowitch J. Infectious Amplification of Wild-Type Human-Immunodeficiency-Virus from Patients Lymphocytes and Modulation by Reverse-Transcriptase Inhibitors in-Vitro. Antimicrob Agents Ch. 1993;37(10):2206–11. WOS:A1993MA17900026. 10.1128/aac.37.10.2206 PMC192251 7504908

[49] Lorenzo-Redondo R, Fryer HR, Bedford T, Kim EY, Archer J, Pond SLK, et al Persistent HIV-1 replication maintains the tissue reservoir during therapy. Nature. 2016;530(7588):51-+. doi: 10.1038/nature16933. WOS:000369304500030. 26814962 10.1038/nature16933 PMC4865637

[50] Fletcher CV, Staskus K, Wietgrefe SW, Rothenberger M, Reilly C, Chipman JG, et al Persistent HIV-1 replication is associated with lower antiretroviral drug concentrations in lymphatic tissues. Proc Natl Acad Sci U S A. 2014;111(6):2307–12. doi: 10.1073/pnas.1318249111 ; PubMed Central PMCID: PMCPMC3926074. 24469825 10.1073/pnas.1318249111 PMC3926074

[51] Cory TJ, Schacker TW, Stevenson M, Fletcher CV. Overcoming pharmacologic sanctuaries. Curr Opin Hiv Aids. 2013;8(3):190–5. doi: 10.1097/COH.0b013e32835fc68a. WOS:000317591500006. 23454865 10.1097/COH.0b013e32835fc68a PMC3677586

