# Supplementary material for: Dendritic cells efficiently transmit HIV to T Cells in a tenofovir and raltegravir insensitive manner
Source: PLoS One. 2018 Jan 2;13(1):e0189945. doi: 10.1371/journal.pone.0189945 (PMC5749731; doi:10.1371/journal.pone.0189945)
Supplement: S1 Fig — (PDF) [file pone.0189945.s001.pdf]

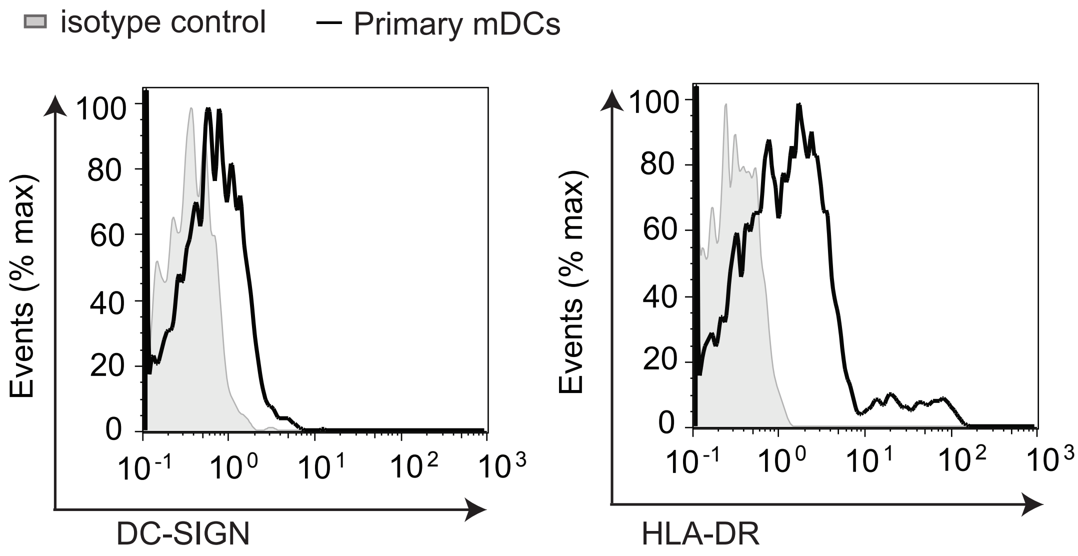

S1 Fig. DC-SIGN and HLA-DR expression of LPS-activated myeloid mDCs.
Pirmary mDCs were treated with LPS and analyzed by flow cytometry. FACS histogram plots showing expression of DC-SIGN and HLA-DR.
