# Supplementary material for: Dendritic cells efficiently transmit HIV to T Cells in a tenofovir and raltegravir insensitive manner
Source: PLoS One. 2018 Jan 2;13(1):e0189945. doi: 10.1371/journal.pone.0189945 (PMC5749731; doi:10.1371/journal.pone.0189945)
Supplement: S1 Table — (PDF) [file pone.0189945.s002.pdf]

S1 Table. HIV-1 molecular clones.

Molecular clone	Tropism	Catalog No.	Source
pNL4-3	CXCR4	114	AIDS Reagent Program
pLAI.2	CXCR4	2532	AIDS Reagent Program
pWT/BaL	CCR5	11414	AIDS Reagent Program
pYK-JRCSF	CCR5	2708	AIDS Reagent Program
NFN-SX	CCR5		(50)
p89.6	dual	3552	AIDS Reagent Program
pWITO.c/2474	CCR5	11739	AIDS Reagent Program
pCH040.c/2625	CCR5	11740	AIDS Reagent Program
pCH077.t/2627	CCR5	11742	AIDS Reagent Program
pCH106.c/2633	CCR5	11743	AIDS Reagent Program
pTHRO.c/2626	CCR5	11745	AIDS Reagent Program
pREJO.c/2864	CCR5	11746	AIDS Reagent Program
pCH058.c/2960	CCR5	11856	AIDS Reagent Program
