# Supplementary material for: Dendritic cells efficiently transmit HIV to T Cells in a tenofovir and raltegravir insensitive manner
Source: PLoS One. 2018 Jan 2;13(1):e0189945. doi: 10.1371/journal.pone.0189945 (PMC5749731; doi:10.1371/journal.pone.0189945)
Supplement: S2 Table — (PDF) [file pone.0189945.s003.pdf]

S2 Table. Antibodies used in this study.

Antibody	Catalog No.	Source
anti-human CD14	HCD14	Biolegend
anti-human CD3	300308	Biolegend
anti-human CD86	IT2.2	Biolegend
Anti-human DC-SIGN	9E9A8	Biolegend
anti-human HLA-DR	L243	Biolegend
anti-p24	KC57	Beckman Coulter
